# A proof of the DBRF-MEGN method, an algorithm for deducing minimum equivalent gene networks

**DOI:** 10.1186/1751-0473-6-12

**Published:** 2011-06-24

**Authors:** Koji Kyoda, Kotaro Baba, Hiroaki Kitano, Shuichi Onami

**Affiliations:** 1 Laboratory for Developmental Dynamics, RIKEN Quantitative Biology Center, and Advanced Computational Sciences Department, RIKEN Advanced Science Institute, 1-7-22 Suehirocho, Tsurumi, Yokohama 230-0045, Japan; 2 Graduate School of Science and Technology, Keio University, 3-14-1 Hiyoshi, Kohoku, Yokohama 223-8522, Japan; 3 Kitano Symbiotic Systems Project, ERATO, Japan Science and Technology Corporation, M31 6A, 6-31-15 Jingumae, Shibuya, Tokyo 150-0001, Japan; 4 The Systems Biology Institute, 5-6-9 Shirokanedai, Minato, Tokyo 108-0071, Japan; 5 National Institute of Agrobiological Sciences, 2-1-2 Kannondai, Tsukuba, Ibaraki 305-8602, Japan; 6 Sony Computer Science Laboratories, Inc, 3-14-13 Higashi-gotanda, Shinagawa, Tokyo 141-0022, Japan

**Keywords:** DBRF-MEGN method, proof, algorithm, gene network, expression profiles

## Abstract

**Background:**

We previously developed the DBRF-MEGN (difference-based regulation finding-minimum equivalent gene network) method, which deduces the most parsimonious signed directed graphs (SDGs) consistent with expression profiles of single-gene deletion mutants. However, until the present study, we have not presented the details of the method's algorithm or a proof of the algorithm.

**Results:**

We describe in detail the algorithm of the DBRF-MEGN method and prove that the algorithm deduces all of the exact solutions of the most parsimonious SDGs consistent with expression profiles of gene deletion mutants.

**Conclusions:**

The DBRF-MEGN method provides all of the exact solutions of the most parsimonious SDGs consistent with expression profiles of gene deletion mutants.

## Background

Identification of gene regulatory networks (hereafter called gene networks) is essential for understanding cellular functions. Large-scale gene deletion projects [1-4] and DNA microarrays [5,6] have enabled the creation of large-scale gene expression profiles of gene deletion mutants [7,8]; these large-scale profiles comprise the expression levels of thousands of genes measured in deletion mutants of those genes. Such profiles are invaluable sources for identifying gene networks. Many procedures have been developed for inferring gene networks from such profiles [9-18].

Kyoda et al. developed the DBRF-MEGN (difference-based regulation finding-minimum equivalent gene network) method, an algorithm for inferring gene networks from large-scale gene expression profiles of gene deletion mutants [14]. In this algorithm, gene networks are modeled as signed directed graphs (SDGs) in which a regulation between two genes is represented as a signed directed edge whose sign - positive or negative - represents whether the effect of the regulation is activation or inhibition and whose direction represents which gene regulates which other gene; the most parsimonious SDGs consistent with the expression profiles are thus deduced. Kyoda et al. showed that the method is applicable to large-scale gene expression profiles of gene deletion mutants and that networks deduced by the method are valid and useful for predicting functions of genes [14]. However, details of the method's algorithm and a proof of the algorithm have not previously been published.

Here we describe in detail the algorithm of the DBRF-MEGN method and prove that the algorithm provides all of the exact solutions of the most parsimonious gene networks consistent with expression profiles of gene deletion mutants.

### Implementation

The software of the DBRF-MEGN method was written in C++ under Linux. The complete source code files, a binary Linux executable file, and the software manual are available [see Additional File 1].

## Results

### Difference-based deduction of initially deduced edges and the minimum equivalent gene networks

The DBRF-MEGN method consists of five processes, namely (1) difference-based deduction of initially deduced edges, (2) removal of non-essential edges from the initially deduced edges, (3) selection of the uncovered edges in main components from the non-essential edges, (4) separation of the uncovered edges in main components into independent groups, and (5) restoration of the minimum number of edges from each independent group [14]. First, we define a gene network modeled as an SDG:

**Definition 1: **A signed directed graph (SDG) is given by a tuple *G *= (*V, E, f*) with a set *V *of nodes (genes), a set *E*⊆*V×V *of directed edges, and an edge sign function *f:E*→{± 1}, which is an integral part of an SDG.

**Figure 1:**
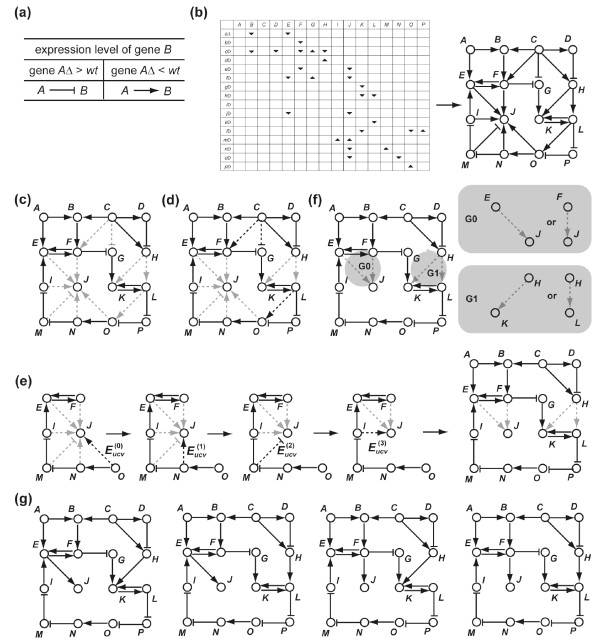
**An example of the deduction of MEGNs from the expression profiles of gene deletion mutants**. **(a) **An assumption used in the DBRF-MEGN method. **(b) **Deduction of the initially deduced edges. The matrix represents a set of expression profiles and the schematic represents a set of initially deduced edges. In the matrix, *A, B*, ... represent expression levels of gene *A*, gene *B*, ..., and *aΔ, bΔ*, ... represent deletion mutants of gene *A, B*, ... The up (down) arrows indicate that the gene expression levels are higher (lower) in the deletion mutant than in the wild type. **(c) **Essential edges. Non-essential edges are gray-dotted. **(d) **Uncovered edges. Uncovered edges are gray-dotted and covered edges are black-dotted. **(e) **Exclusion of uncovered edges in peripheral components. , , , and  are uncovered edges in peripheral components. The resulting four gray-dotted edges are uncovered edges in main components. **(f) **Independent groups of uncovered edges in main components. For each group, the minimum number of edges with which essential edges can explain all edges in the group are shown: (*E, J*) or (*F, J*) for G0, and (*H, K*) or (*H, L*) for G1. **(g) **Four MEGNs of the profiles. Combinations of the minimum numbers of edges of two independent groups (G0 and G1) produce all four MEGNs.

The first process of the DBRF-MEGN method is "difference-based deduction of initially deduced edges" (Figure 1b), which uses an assumption that is commonly made in genetics and cell biology [14], i.e., there exists a positive (negative) regulation from gene *A *to gene *B *when the expression level of gene *B *in the deletion mutant of gene *A *is significantly lower (higher) than in the wild-type (Figure 1a). For each possible pair of genes in the profiles, the process determines whether positive (negative) regulations between those genes exist and deduces all edges consistent with both the assumption and the profiles by detecting the difference in expression levels between the wild type and deletion mutants; we call these edges *initially deduced edge*s.

**Definition 2: **Let us assume the intervention experiments have been performed for the gene set *J, J *⊆ *V*. Let *D *= (d*_jk_*) ∈*R^J×V ^*be a matrix such that *d_jk _*represents the expression of gene *k *after an intervention in gene *j *(relative to wild-type expression). From this, we deduce the graph initially deduced edges, *G_ide _= (V,E_ide_,f)*. We assume a negative regulation of *k *by *j *if *d_jk _*> α for some suitably chosen constant α. Analogously, a positive regulation of *k *by *j *is postulated whenever *d_jk _*<*β *for some *β *(sensibly, we require *β *< 0 < α). Formally,

and *f:E_ide_*→{± 1} is given by *f*((*j,k*)) = 1 if there is a positive regulation of *k *by *j*, and otherwise *f*((*j,k*)) = -1.

The thresholds α and β determine the significance of the difference in expression levels between the wild type and deletion mutants. These thresholds can be specified by various procedures such as by using fold-change or the statistical significance of the expression level [7,8,14,19,20].

**Figure 2:**
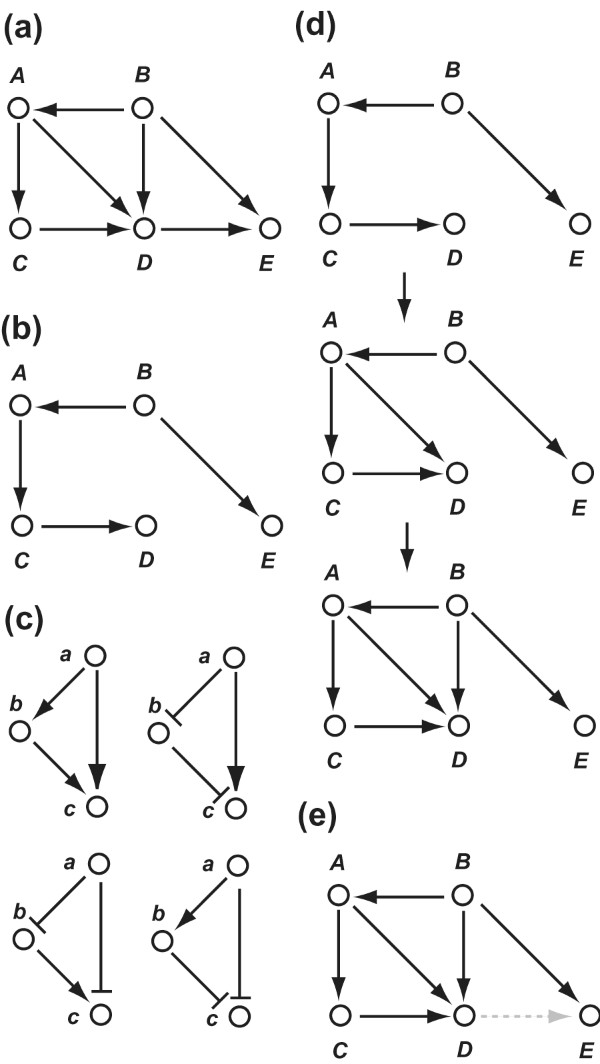
**Introduction to the function "exp" and the concept "cover"**. **(a) **Initially deduced edges (*E_ide_*). **(b) **A set of edges (*E_p_*). **(c) **Four cases of *exp*(*a, b, c*) = 1. In each case, (*a, c*) is *explained *by (*a, b*) and (*b, c*). **(d) **The set of edges that are covered by edges in *E_p _*(). (*A, C*) and (*C, D*) explain (*A, D*); the (*A, D*) and (*B, A*) explain (*B, D*). Thus, (*A, D*) and (*B, D*) are *covered *by edges in *E_p_*. (*D, E*) is not *covered *by edges in *E_p _*because (*D, E*) cannot be explained by edges in *E_p_*. **(e) **Edges that are covered by edges in *E_p _*(black) and those that are not covered by edges in *E_p _*(gray-dotted).

The DBRF-MEGN method deduces the most parsimonious SDGs consistent with the SDG that consists of the initially deduced edges. Before defining the most parsimonious SDGs, we need to introduce the function *exp *and the concept *cover *(Figure 2).

**Definition 3: **If, and only if, ∃ (*i, j*), (*j, k*), (*i, k*) | *f*(*i, j*) × *f*(*j, k*) = *f*(*i, k*), then *exp*(*i, j, k*) = 1; otherwise, *exp*(*i, j, k*) = 0.

**Definition 4: **Let *E_p _*⊆ *E_ide _*be a set of edges. Define  and by induction  such that *exp*(*j*,*i*,*k*)=1}. Moreover, let .

**Remark: **The family of edge sets on *V *is partially ordered by set inclusion. If *E*_1_⊆*E*_2_, note that by a trivial induction on *r*, , and hence . This means that the mapping  is monotonic. Let *E *⊆ *E_ide_*. By construction, an edge (*j, k*) from  is an element of  for suitable *r,s *∈ *N*. This implies . Thus , and the mapping  is a so-called closure operation.

**Lemma 1: **If *E*_1_⊆*E*_2_, .

**Proof: **The remark proves lemma 1.

**Lemma 2: **If , then .

**Proof: ** by monotonicity and closure of the mapping .^cov^.

**Lemma 3: **If  and , then .

**Proof: **By  by monotonicity and closure of the mapping.^cov^.

**Figure 3:**
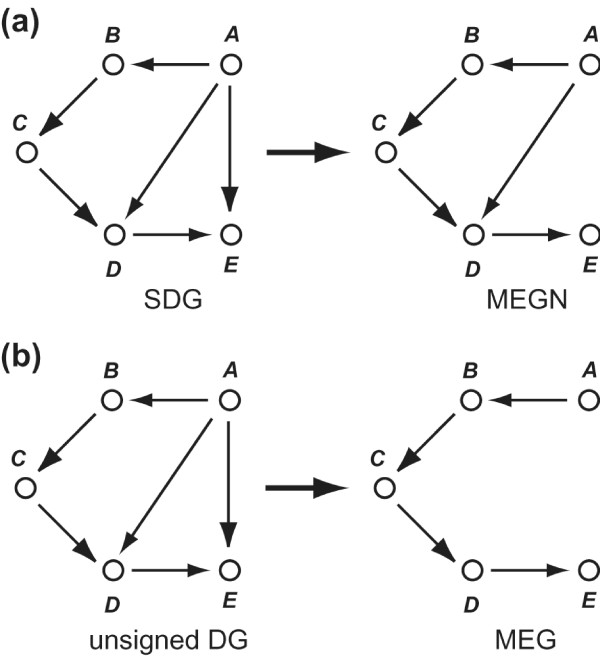
**Difference between MEGN and MEG**. Deduction of the MEGN **(a) **and the MEG **(b) **from the same graph is shown. The MEGN includes the edge from *A *to *D *because no two edges explain the edge. In contrast, the MEG does not include the edge from *A *to *D *because *A *can reach *D *without using the edge from *A *to *D *(*A→B→C→D*). The MEGN consists only of the essential edges.

Now, we define the most parsimonious SDGs consistent with the expression profiles of gene deletion mutants. A most parsimonious SDG consists of the minimum number of edges that "cover" all initially deduced edges. By this definition, an edge can be redundant only when it is "explained" by two other initially deduced edges. Importantly, an edge is not redundant when it is "explained" by only three or more initially deduced edges (Figure 3a). We call the most parsimonious SDGs *minimum equivalent gene networks *(*MEGNs*).

**Definition 5: ** (where  is the restriction of *f *to *E*_0_) is a most parsimonious SDG, named a MEGN, of *G *= (*V,E_ide_,f*) if and only if it satisfies the following conditions: (1) *E*_0_⊆*E_ide_*, (2) , (3) ∀ *E_p _*⊆ *E_ide _*such that , . Since we keep *G *= (*V,E_ide_,f*) fixed for the rest of the paper, we often call *G*_0 _simply a MEGN, without explicit reference to *G*.

### Removal of non-essential edges from the initially deduced edges

The second process of the DBRF-MEGN method removes all non-essential edges from the initially deduced edges. The process removes all edges that are explained by two other initially deduced edges (Figure 1c). The resulting edges are called *essential edges *and the removed edges are called *non-essential edges*.

**Definition 6: **If there exist (*i, j*), (*j, k*), (*i, k*) ∈ *E_ide _*such that *exp*(*i, j, k*) = 1, then (*i, k*) is called a *non-essential edge*. Let *E_nes _*be the set of non-essential edges. The set *E_es _*of essential edges is the complement of *E_nes _*in *E_ide_, E_es _*= *E_ide_*\*E_nes_*.

Essential edges and non-essential edges have the following properties.

**Lemma 4: **If *E_p _*⊆ *E_ide _*and , then *E*_*p *_⊇ *E*_*es*_.

**Proof: **Assume that there exists (*i, j*) ∈*E_es _*such that (*i, j*)  and (*i, j*) ∉ *E_p_*. Because (*i, j*)  and (*i, j*) ∉ *E_p_*, there exist (*i, k*), (*k, j*) such that *exp*(*i, k, j*) = 1. This contradicts our assumption (*i, j*) ∉ *E_es_*.

**Lemma 5: **If  is a MEGN, *E_es _*⊆ *E*_0_.

**Proof: **, hence *E_0 _*⊇ *E_es _*by lemma 4.

When the essential edges cover all initially deduced edges, the SDG consisting of the essential edges is the only MEGN consistent with the profiles.

**Theorem 1: **If , then  is the unique MEGN of *G *= (*V, E_ide_, f*).

**Proof: **By hypothesis, conditions (1) *E_es _*⊆ *E_ide_*, and (2) , of a MEGN are met. It remains to show the uniqueness and minimality of *E_es_*. (3) Let  be an arbitrary MEGN. Then by lemma 5, *E_es _*⊆ *E*_0_, and by minimality of *E*_0_, it follows that *E_es _*= *E*_0_. The theorem is proved.

### Selection of the uncovered edges in main components from the non-essential edges

The essential edges sometime fail to cover all initially deduced edges because some edges in the initially deduced edges represent direct gene regulations even when they are explained by two other edges (Figure 1d). In this case, the method restores the minimum number of non-essential edges so that the resulting edges (essential edges and the restored non-essential edges) cover all initially deduced edges. The SDG, consisting of essential edges and of the restored non-essential edges, is a MEGN. Before selecting the sets of non-essential edges to be restored, the method distinguishes non-essential edges that have a chance to be included in the MEGNs from those that do not in order to reduce the number of non-essential edges to be considered for the restoration and thus to reduce the computational cost to find non-essential edges to be restored. This third process of the DBRF-MEGN method consists of two sub-processes, namely (a) selection of uncovered edges and (b) selection of uncovered edges in main components. The resulting non-essential edges are called *uncovered edges in main components*, and from these edges the later processes of the DBRF-MEGN method select edges that are included in the MEGNs.

#### a) Selection of uncovered edges

The first sub-process distinguishes the non-essential edges that are covered by the essential edges from those that are not (Figure 1d). Those edges are called *covered edges *and *uncovered edges*, respectively.

**Definition 7: **Let *E_cv _*= (*E_es_*)^cov^\ *E_es _*be the set of *covered *edges. Let *E_ucv _*= *E_ide _*\( *E_es _*∪ *E_cv_*) be the set of *uncovered *edges. The set of initially deduced edges is thereby partitioned into three disjoint edge sets: *E_ide _*= *E_es _*∪ *E_cv _*∪ *E_ucv_*.

Here, we prove that the MEGNs do not include covered edges.

**Lemma 6: **If  is a MEGN, then *E_es _*⊆ *E_0 _*⊆ *E_es _*∪ *E_ucv_*.

**Proof: **First, *E_es _*⊆ *E_0 _*by lemma 5. By definition 7, *E_es _*⊆ *E_0_\E_cv_*, hence  by monotonicity of.^cov^. It follows that  by lemma 2. By minimality of *E*_0_, *E_0 _*= *E_0_\ E_cv_*, which is equivalent to *E_0 _*∩ *E_cv _*= *Φ*. By definition 7, this implies *E_0 _*⊆ *E_es _*∪ *E_ucv_*, completing the proof.

#### b) Selection of uncovered edges in main components

**Figure 4:**
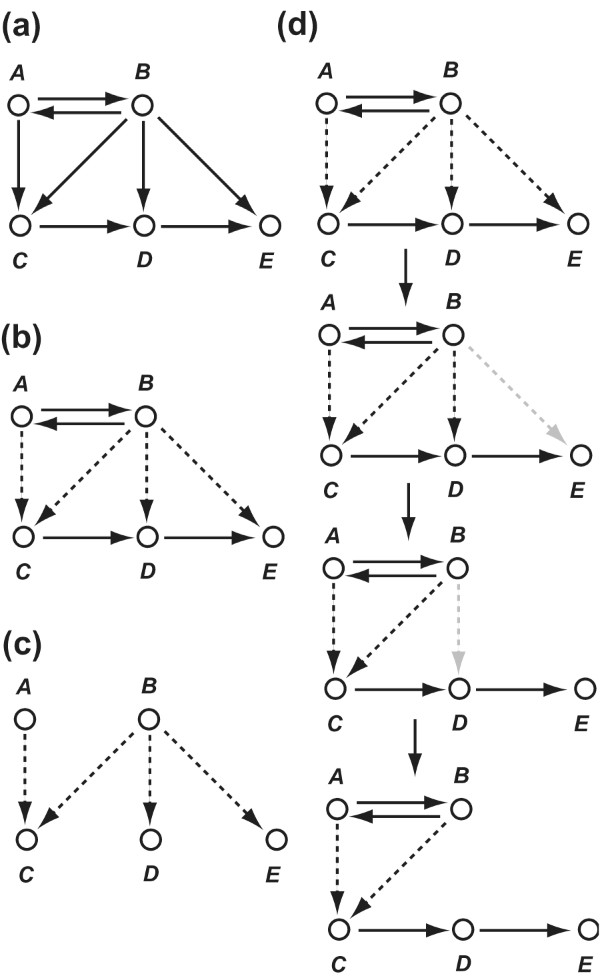
**Example of uncovered edges in peripheral and main components**. **(a) **Initially deduced edges (*E_ide_*). **(b) **Essential edges (*E_es_*). Non-essential edges are dotted. **(c) **Uncovered edges (*E_ucv_*). Because all non-essential edges cannot be covered by essential edges, the non-essential edges are called *uncovered edges *(*E_ucv_*). **(d) **Uncovered edges in peripheral components (gray-dotted). (*B, D*) and (*B, E*) are uncovered edges in peripheral components because (*B, E*) does not explain any other edges in *E_ucv _*with an edge in *E_ide_*, and (*B, D*) does not explain any other edges in *E_ucv _*except (*B, E*) with an edge in *E_ide_*. (*A, C*) and (*B, C*) are uncovered edges in main components. If edges in a MEGN cover (*A, C*) and (*B, C*), the edges also cover (*B, D*) and (*B, E*). (*B, D*) and (*B, E*) cover no edges except themselves. Thus, (*B, D*) and (*B, E*) are not included in the MEGNs.

The second sub-process distinguishes uncovered edges that have a chance to be included in the MEGNs from those that do not (Figure 1e; Figure 4). Those edges are called uncovered edges in main components and *uncovered edges in peripheral components*. The uncovered edges in peripheral components are defined as follows:

**Definition 8: **Define  be the set of uncovered edges (*i,j*) ∈ *E_ucv _*which cannot be used to directly explain another uncovered edge in *E_ucv _*with the other edges (*k,i*) ∈ *E_ide _*or (*j,k*) ∈ *E_ide_*.

**Lemma 7: **.

**Proof: **By definition 8, the edges in  cannot explain another uncovered edges in *E_ucv_*. Therefore, the edges in  can be explained by the edges in . The lemma is proved.

**Definition 9: **Following the definition 8, define  which cannot be used to directly explain another uncovered edge in  with the other edges (*k,i*) ∈ *E_ide _*or (*j,k*) ∈ *E_ide_*}. Let  be the set of *uncovered edges in peripheral components*. Let  be the set of *uncovered edges in main components*. The set of initially deduced edges is thereby partitioned into four disjoint edge sets: .

In the following, we prove that the MEGNs do not include uncovered edges in peripheral components. First, we prove that uncovered edges in peripheral components have the following properties.

**Lemma 8: **.

**Proof: **We prove lemma 8 by mathematical induction. (1) By lemma 7, lemma 8 is true when *r *= 0. By definitions 8 and 9, , hence . By lemmas 2 and 7, . Thus, lemma 8 is true when *r *= 1. (2) Assume that lemma 8 is true when *r *= *m*. This means that we assume that  (2a). By definition 9,  (2b). Because  and (2b),  (2c). Because (2a), (2c) and lemma 3,  (2d). Because (2b) and (2d), . Thus, lemma 8 is true when *r *= *m *+1, if it is true when *r *= *m*. By (1) and (2), lemma 8 is true.

**Lemma 9: **.

**Proof: **By lemma 8, . Because , lemma 9 is true.

Now we prove that the MEGNs do not include uncovered edges in peripheral components.

**Lemma 10: **If  is a MEGN, .

**Proof: **Assume that there exists . Because of lemma 5 and definition 7, , hence  by lemma 6. By the assumption  and definition 8, , hence . By lemmas 2 and 9, . This contradicts our assumption that  is a MEGN. Therefore, . By definition 9 and lemma 6, this implies , completing the proof.

### Separation of the uncovered edges in main components into independent groups and restoration of the minimum number of edges from each independent group

**Figure 5:**
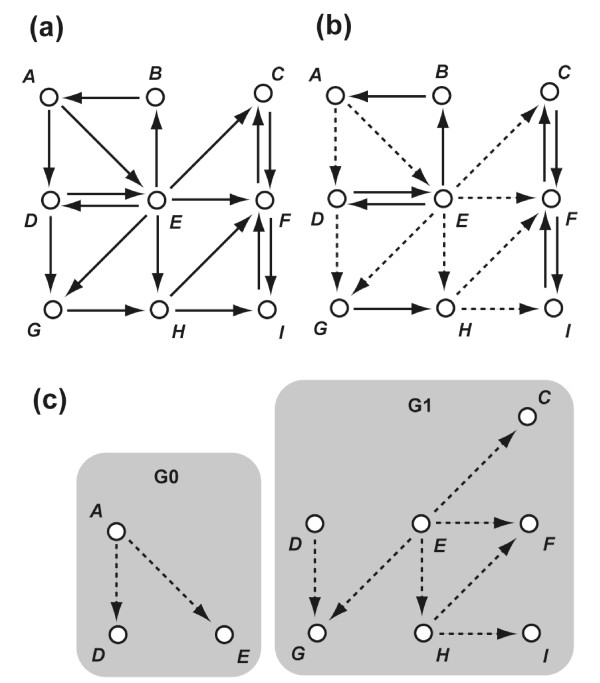
**An example of independent groups**. **(a) **Initially deduced edges (*E_ide_*). **(b) **Essential edges (*E_es_*). Uncovered edges in main components () are dotted. **(c) **Independent groups of uncovered edges in main components. Uncovered edges in main components are separated into two independent groups G0 () and G1 (). Edges in one group do not explain those in other group.  consists of  and .  consists of , , , , and .

The fourth process of the DBRF-MEGN method separates uncovered edges in main components into "independent groups" so that edges to be restored can be deduced independently for each group (Figure 1f; Figure 5). For each group, the fifth process of the DBRF-MEGN method deduces the minimum number of edges with which essential edges can cover all edges in the group. All sets of such edges are deduced for each group. The essential edges and any possible combination of these sets from each group generate a MEGN of the profiles (Figure 1g).

The independent groups are generated so that the edges in one group do not cover those in other groups.

**Definition 10: **Define  be a set of an edge , and by induction  such that *exp*(*i, j, k*) = 1 or  such that *exp*(*k, i, j*) = 1 or  such that *exp*(*i, k, j*) = 1 or  such that *exp*(*i, k, j*) = 1}. Let  be the set of edges in an *independent group*. Let , where  is a set of an edge  and by induction  such that *exp*(*i, j, k*) = 1 or  such that *exp*(*k, i, j*) = 1 or  such that *exp*(*i, k, j*) = 1 or  such that *exp*(*i, k, j*) = 1}. Then, .

The essential edges and a combination of sets of the minimum number of edges for each independent group generate a MEGN of the profiles.

**Definition 11: **Let  be the set of edges in *i*th independent group that satisfies (1) , (2) , and (3)  such that , .

We prove that the essential edges and a combination of sets of the minimum number of edges for each independent group generate a MEGN of the profiles as follows:

**Lemma 11: **If there exist (*i, j*) ∈ , (*i, k*), (*k, j*) ∈ *E_ide _*such that *exp*(*i, k, j*) = 1, then {(*i, k*), (*k, j*)} ∩ *E_ucv _*⊆ .

**Proof: **By definition 10, lemma 11 is true.

**Lemma 12: **.

**Proof: **By definitions 7 and 11, . Because , . By lemmas 2 and 8, . Therefore, .

**Theorem 2: ** is a MEGN.

**Proof: **(1)  by the condition of theorem 2.(2) By lemma 12, . (3) By lemmas 4, 11 and definition 11, ∀ *E_p _*⊆ *E_ide _*such that , . Because  and lemma 2, ∀ *E_p _*⊆ *E_ide _*such that , . The theorem is proved.

**Remark: **When there exist more than one solution of the minimum number of edges for independent groups, the SDGs each of which consists of the essential edges and a possible combination of sets of the solutions for each independent group are MEGNs because these SDGs must satisfy the conditions in definition 5.

### Algorithms of the DBRF-MEGN method

We are concerned with algorithms that are computationally efficient for deducing MEGNs from expression profiles of single-gene deletion mutants. We list these in a form easily translatable into a computer program.

#### (A1) Algorithm for deducing initially deduced edges

**double ***d*[*n*][*n*]: gene expression profiles

**int ***t*[*n*][*n*]

**void ***dbrf*()

  **int ***i, j*;

  **for ***i *= 1 **to ***n ***do**

    **for ***j *= 1 **to ***n ***do**

      **if ***d*[*i*][*j*] <*β *&*i *≠ *j ***then**

        *t*[*i*][*j*]: = +1;

      **else if ***d*[*i*][*j*] > α &*i *≠ *j ***then**

        *t*[*i*][*j*]: = -1;

      **else**

        *t*[*i*][*j*]: = 0;

The matrix *d*[*n*][*n*] represents the gene expression profiles. Each entry *d*[*i*][*j*] represents the log-ratio of the expression of gene *j *in gene *i *deletion mutants to that in the wild-type. The non-zero entries of the resulting matrix *t*[*n*][*n*] represent the initially deduced edges. If an entry *t*[*i*][*j*] is +1 or-1, it represents a positive or negative edge from gene *i *to gene *j*, respectively. The number of complete iterations is bounded by *n^2^*.

#### (A2) Algorithm for distinguishing the essential edges from the non-essential edges

**int ***t*[*n*][*n*]: initially deduced edges

**void ***ess_noness*()

  **int ***i, j, k*;

  **for ***j *= 1 **to ***n ***do**

    **for ***i *= 1 **to ***n ***do**

    **if ***t*[*i*][*j*] ≠ 0 **then**

      **for ***k *= 1 **to ***n ***do**

        **if ***t*[*j*][*k*] ≠ 0 &*t*[*i*][*k*] ≠ 0 &*t*[*i*][*k*] = *t*[*i*][*j*]× *t*[*j*][*k*] **then**

          *check*(*t*[*i*][*k*]);

The checked entries of the matrix *t*[*n*][*n*] represent non-essential edges. The unchecked non-zero entries of the resulted matrix *t*[*n*][*n*] represent essential edges. We created this algorithm by modifying Warshall's algorithm [21]. The number of complete iterations is bounded by *n^3^*.

#### (A3.1) Algorithm for distinguishing uncovered edges from covered edges

**int ***t*[*n*][*n*]: initially deduced edges

**int ***e*[*n*][*n*]: essential edges

  **void ***covered_edge*()

    **int ***i, j, k*;

    **bool ***finished*;

    *finished *: = *false*;

    **while ***finished *= *false ***do**

      *finished *: = *true*;

      **for ***i *= 1 **to ***n ***do**

        **for ***j *= 1 **to ***n ***do**

          **if ***e*[*i*][*j*] ≠ 0 **then**

            **for ***k *= 1 **to ***n ***do**

              **if ***e*[*j*][*k*] ≠ 0 &*t*[*i*][*k*] ≠ 0 &*t*[*i*][*k*] = *e*[*i*][*j*] × *e*[*j*][*k*] **then**

                *e*[*i*][*k*]: = *t*[*i*][*k*];

                *check*(*e*[*i*][*k*]);

                *finished *: = *false*;

The checked entries of the matrix *e*[*n*][*n*] represent covered edges. The non-zero entries of the matrix *t*[*n*][*n*] that differ from the non-zero entries of the resulted matrix *e*[*n*][*n*] represent uncovered edges. This algorithm iterates over the while loop to find edges in *E_nes _*that can be covered by the essential edges. Thus, the number of iterations is bounded by .

#### (A3.2) Algorithm for finding uncovered edges in peripheral components

**int ***t*[*n*][*n*]: initially deduced edges

**int ***u*[*n*][*n*]: uncovered edges

**void ***peripheral_uncovered*()

  **int ***i, j, k*;

  **bool ***flag, sflag*;

  *flag *: = *false*;

  **while ***flag *= *false ***do**

    *flag *: = *true*;

    **for ***j *= 1 **to ***n ***do**

    **for ***i *= 1 **to ***n ***do**

      **if ***u*[*i*][*j*] ≠ 0 **then**

      *sflag *: = *false*;

      **for ***k *= 1 **to ***n ***do**

        **if ***t*[*j*][*k*] ≠ 0 &*u*[*i*][*k*] ≠ 0 &*t*[*i*][*k*] = *t*[*i*][*j*] × *t*[*j*][*k*] **then**

        *sflag *: = *true*;

        **if ***t*[*k*][*i*] ≠ 0 &*u*[*k*][*j*] ≠ 0 &*t*[*k*][*j*] = *t*[*k*][*i*] × *t*[*i*][*j*] **then**

        *sflag *: = *true*;

        **if ***sflag *= *false ***then**

          *check*(*u*[*i*][*j*]);

          *flag *: = *false*;

          *rm*_*checked*_*edge*(); // set all checked entries to 0

The entries of the resulted matrix *u*[*n*][*n*] that have been changed from +1 or -1 to 0 represent uncovered edges in peripheral components. The non-zero entries of the resulted matrix *u*[*n*][*n*] represent uncovered edges in main components. This algorithm iterates over the while loop to find edges in *E_ucv_*that are to be included in . Thus, the number of complete iterations is bounded by .

#### (A4.1) Algorithm for dividing uncovered edges in main components () into independent groups

**int ***t*[*n*][*n*]: initially deduced edges

**int ***e*[*n*][*n*]: uncovered edges in main components

**ig ***indgrp *: independent group

**list **<*edge *>*el *: edge list

**list **<*ig *>*igl *: independent group list

**void ***independent_group*()

  **int ***i, j*;

  **for ***i *= 1 **to ***n ***do**

    **for ***j *= 1 **to ***n ***do**

      **if ***e*[*i*][*j*] ≠0 **then**

        *el*.*clear*();

        *el*.*append*(*e_ij_*);

        *append*_*group*(*i, j*);

        *indgrp.init*();

        *indgrp*.*set_el*(*el*);     // store edge list *el *in *indgrp*

        *igl*.*append*(*indgrp*);     // *indgrp *: an independent group

**void ***append_group*(**int ***i*, **int ***j*)

  **int ***x*;

  **for ***x *= 1 **to ***n ***do**

    **if ***t*[*i*][*x*] ≠0 &*t*[*x*][*j*] ≠0 **then**

    **if ***t*[*i*][*j*] = *t*[*i*][*x*] × *t*[*x*][*j*] **then**

      **if ***e*[*i*][*x*] ≠0 **then**

      *el*.*append*(*e_ix_*);

      *e*[*i*][*x*]: = 0;

      *append*_*group*(*i, x*);

    **if ***e*[*x*][*j*] ≠0 **then**

      *el*.*append*(*e_xj_*);

      *e*[*x*][*j*]: = 0;

      *append*_*group*(*x, j*);

    **if ***t*[*x*][*i*] ≠0 &*t*[*x*][*j*] ≠0 **then**

    **if ***t*[*x*][*j*] = *t*[*x*][*i*] × *t*[*i*][*j*] **then**

      **if ***e*[*x*][*j*] ≠0 **then**

      *el*.*append*(*e_xj_*);

      *e*[*x*][*j*]: = 0;

      *append*_*group*(*x, j*);

    **if ***t*[*j*][*x*] ≠0 &*t*[*i*][*x*] ≠0 **then**

    **if ***t*[*i*][*x*] = *t*[*i*][*j*] × *t*[*j*][*x*] **then**

      **if ***e*[*i*][*x*] ≠0 **then**

      *el*.*append*(*e_ix_*);

      *e*[*i*][*x*]: = 0;

      *append*_*group*(*i, x*);

The number of complete iterations of *independent*_*group*() is bounded by *n*^2^. The number of complete iterations of *append*_*group*(*int, int*) is bounded . Thus, the number of complete iterations is bounded by .

#### (A4.2) Algorithm for finding all sets of minimum number of edges to be restored in each independent group

**int ***e*[*n*][*n*]: essential edges and uncovered edges in peripheral components

**ig ***indgrp *: independent group

**list **<*ig *>*igl *: independent group list

**list **<*edge *>*el, tmp*_*el *: edge list

**list **<*edge list *>*combi_el *: combination of edge list

  **void ***find_min_ig*()

    **int ***i, num*_*edge*;

    **for ***i *= 1 to *igl*.*size*() do

      *combi*_*el*.*clear*(); *el*.*clear*();

      *indgrp *← *igl*.*get*_*ig*(*i*);     // copy the *i*th independent group from *igl*

      *el *← *indgrp*.*get*_*el*();

      **for ***num*_*edge *= 1 **to ***el*.*size*() **do**

      *add*_*edge*(*num*_*edge*, 1);

      **if **(*combi*_*el*.*size*() > 0) **then**

        **break;**

      *set*_*min*_*combi*_*el*(*i, combi*_*el*);     // store *combi*_*el *in the *i*th independent group

  **void ***add*_*edge*(**int ***num*_*edge*, **int ***start*)

    **int ***j*;

    **if ***start *+ *num*_*edge *- 1 >*el*.*size*() **then**

      **return;**

    **for ***j *= *start ***to ***el*.*size*() **do**

    *set*_*edge*(*j*);     // set the entry of *e*[*n*][*n*] corresponding to the *j*th edge

            // in *el *to +1 or -1 according to the sign of the edge

    *check*(*el*.*get*_*edge*(*j*));     // check the *j*th edge in *el*

    **if ***num*_*edge *> 1 **then**

    *add*_*edge*(*num*_*edge *- 1, *j *+ 1);

else

    **if **(*confirm*() = *true*) **then**

      *tmp*_*el*.*clear*();

      *set*_*tmp*_*el*();     // append all checked edges to *tmp*_*el*

      *combi*_*el*.*append*(*tmp*_*el*);     // *tmp*_*el *: a set of the minimum number of

            // edges to be restored

    *reset*_*edge*(*j*);   // set the entry of *e*[*n*][*n*] corresponding to the *j*th edge

            // in *el *to 0

    *uncheck*(*el.get_edge*(*j*));     // uncheck the *j*th edge in *el*

**bool ***confirm*(): when resulting edges *e*[*n*][*n*] can covered all edges in the group, return *true*.

The number of complete iterations is bounded by , where *G *is the number of independent groups, *R_j _*is the number of edges in the *j*th independent group, *n_j _*is the number of genes in the *j*th independent group, and *m_j _*is the number of edges to be restored in the *j*th independent group.

#### (A5) Algorithm for deducing all MEGNs by making all possible combinations of sets of the minimum number of edges for each independent group

**int ***e*[*n*][*n*]: essential edges

**int ***megn *: the number of MEGNs

**list **<*ig *>*igl *: independent group list

**list **<*edge list *>*tmp*_*combi*_*el *: combination of edge list

**list **<*edge *>*tmp*_*el *: edge list

**void ***megn*()

    **int ***i*;

    *i *: = 1; *megn *: = 0;

    *sub*_*megn*(*i*);

    **if ***megn *= 0 **then**

      *e*[*n*][*n*]: MEGN     // *e*[*n*][*n*] represents the MEGN when 

**void ***sub*_*megn*(**int ***i*)

    **int ***x, y, count*;

    **if ***i *>*igl*.*size*() **then**

      **return;**

*tmp*_*combi*_*el *← *get*_*min_combi*_*el*(*i*);     // copy *combi_el *of the *i*th independent

// group

**for ***y *= 1 **to ***tmp*_*combi*_*el*.*size*() **do**

    *tmp*_*el *← *tmp*_*combi*_*el*.*get*_*el*(*y*);     // copy the *y*th edge list of *tmp*_*combi*_*el*

    *set*_*edges*(*tmp*_*el*);     // set the entries of *e*[*n*][*n*] corresponding to the edges in

        // *tmp*_*el *to +1 or -1 according to the signs of the edges

    **if ***i *= *igl*.*size*() **then**

      *megn*++;

      *e*[*n*][*n*]: MEGN     // *e*[*n*][*n*] represents a MEGN when 

    **else**

      *sub*_*megn*(*i *+ 1)

    *reset*_*edges*(*tmp*_*el*);     // set the entries of *e*[*n*][*n*] corresponding to the edges in

            // *tmp*_*el *to 0

The number of complete iterations is bounded by , where *S_j _*is the number of sets of minimum number of edges to be restored for the *j*th independent group.

## Discussion

We have described in detail the algorithm of the DBRF-MEGN method and have proved that the algorithm provides all of the exact solutions of the most parsimonious gene networks consistent with expression profiles of gene deletion mutants. The resulting gene networks, called MEGNs, are the most parsimonious SDGs consistent with an SDG that consists of the initially deduced edges. In graph theory, many algorithms have been developed for deducing the most parsimonious unsigned directed graphs consistent with a given unsigned directed graph; these graphs are called minimum equivalent graphs (MEGs) [22-25]. MEGN is not just an "SDG version" of MEG, as is explained below. Although both MEGN and MEG are the most parsimonious graphs of a given graph, the parsimoniousness of the graph is defined differently between these graphs. MEGN consists of the minimum number of edges that *cover *all edges of a given graph (initially deduced edges), whereas MEG consists of the minimum number of edges that *retain the reachability *of a given graph [22]. MEGNs use the cover instead of the reachability because a MEGN is a prediction of a gene network consisting only of direct gene regulations [14]. When positive regulations from gene *A *to gene *B*, from gene *B *to gene *C*, from gene *C *to gene *D*, and from gene *A *to gene *D *are detected and regulation from gene *A *to gene *C *is not detected, the regulation from gene *A *to gene *D *is likely to be a direct regulation instead of an indirect regulation as a result of the other three regulations (Figure 3a). The use of cover makes MEGNs include edges representing such likely direct regulations (Figure 3a). In contrast, the MEGs, using reachability, do not include those edges (Figure 3b). Therefore, the DBRF-MEGN method, which deduces MEGNs, is fundamentally different from algorithms that deduce MEGs or algorithms for transitive reduction of SDG [16-18].

The selection of uncovered edges in main components (the third process) and the generation of independent groups (the fourth process) make the DBRF-MEGN method applicable to large-scale gene expression profiles. Without these processes, the computational cost for finding all sets of non-essential edges to be included in the MEGNs is  where *n *is the number of genes and *m *is the number of non-essential edges to be included in a MEGN. This computation is impractical for large-scale gene expression profiles because  increases rapidly as  or *m *increase. The selection of uncovered edges in main components reduces the computational cost to  and the generation of independent groups further reduces it to , where *t *is the number of independent groups, *n_j _*is the number of genes in the *j*th independent group, and *m_j _*is the number of edges in the *j*th independent group to be included in a MEGN.  and *m_j _*are usually far smaller than  and *m*. Because of these reductions of the computational cost, the DBRF-MEGN method successfully deduced MEGNs from sets of large-scale gene expression profiles [14] [see Additional file 2, Table S1; Additional file 3]. Although there is no guarantee that the method will deduce MEGNs from any given expression profiles in an acceptable time, the method would most probably deduce MEGNs from most sets of expression profiles in an acceptable time.

Because MEGNs are deduced from initially deduced edges, the accuracy of MEGNs depends on that of initially deduced edges. The primary source for the inaccuracy in initially deduced edges is the noise of the expression profiles. Importantly, the number of false-positive edges in MEGN depends more on that of falsely-detected edges than that of falsely-missed edges in initially deduced edges; the number of false-negative edges in MEGN depends more on that of falsely-missed edges than that of falsely-detected edges in initially deduced edges [see Additional file 2, Table S2; Additional file 2, Figure S1]. These dependencies suggest the following guideline for the thresholds α and β (Definition 2): when the number of false-positive edges is more important than that of false-negative edges in MEGN, α (β) should be a little higher (lower) than the optimal value; in contrast, when the number of false-negative edges is more important than that of false-positive edges in MEGN, α (β) should be a little lower (higher) than the optimal value.

The DBRF-MEGN method is applicable not only to gene expression profiles of deletion mutants but also to those of gene overexpressions and conditional knock-downs/knock-outs [26-28]. We cannot obtain gene expression profiles of deletion mutants for essential genes. Thus, the method cannot deduce gene networks including essential genes when we use gene expression profiles of deletion mutants. A possible solution for this problem is to use the expression profiles of gene overexpressions or conditional knock-downs/knock-outs. Applications of the DBRF-MEGN method to those profiles will deduce gene regulations that cannot be deduced from gene expression profiles of gene deletion mutants.

A limitation of the DBRF-MEGN method is its inability to deduce (1) self-regulation of genes, and (2) combinatorial gene regulations such as regulation in which the expression of gene *A *is down-regulated only when both gene *B *and gene *C *are inactive. Self-regulation could be deduced by using chromatin immunoprecipitation [29]. Combinatorial gene regulations could be deduced by using the expression profiles of multiple gene deletion mutants [30]. Synthetic genetic arrays can systematically construct a collection of double-gene deletion mutants [31]. A combination of the DBRF-MEGN method and the above techniques would provide more accurate information about gene networks.

When the DBRF-MEGN method is applied to gene expression profiles measured by using DNA microarray, each of the deduced edges represents regulation of one gene's mRNA level by another gene's activity. Therefore, the deduced MEGNs do not include edges that represent post-transcriptional gene regulations although they play major roles in the cell. However, because the algorithm of the DBRF-MEGN method is based on logic that is most commonly used in genetics and cell biology to infer gene networks from small-scale experiments, we can predict post-transcriptional modulators of transcriptional activity from those MEGNs. We predicted total 72 transcriptional regulators and 232 post-transcriptional modulators of 18 transcriptional regulators from the MEGNs deduced from a set of gene expression profiles for 265 *Saccharomyces cerevisiae *genes [14]. The DBRF-MEGN method is applicable not only to gene expression profiles measured by using DNA microarray but also to those measured by using other technologies such as 2D-PAGE-MS [32] and protein chips [33]. MEGNs deduced from those non-DNA microarray expression profiles will include edges that represent post-transcriptional gene regulations in the cell.

## Conclusions

We described in detail the processes of the DBRF-MEGN method and proved that these processes provide all of the exact solutions of the most parsimonious gene networks consistent with the expression profiles of gene deletion mutants, which are called MEGNs. The DBRF-MEGN method provides invaluable information for understanding cellular functions.

## Availability and requirements

Project name: DBRF-MEGN

Project home page: http://so.gsc.riken.jp/dbrf-megn

Operating system: Linux

Programming language: C++

Other requirements: None

Licence: GNU LGPL

Any restrictions to use by non-academics: Licence required

## List of abbreviations

DBRF: difference-based regulation finding; MEGN: minimum equivalent gene network; SDG: signed directed graph; MEG: minimum equivalent graph.

## Competing interests

The authors declare that they have no competing interests.

## Authors' contributions

KK participated in this study's conception, designed the algorithm, proved the algorithm, and drafted the manuscript. KB implemented the algorithm. HK participated in algorithm design. SO conceived this study, designed the algorithm, proved the algorithm, and drafted the manuscript. All authors read and approved the final manuscript.

## Supplementary Material

Additional file 1 **The complete source code files, a binary Linux executable file, and the software manual**. Click here for file

Additional file 2 **Supporting text for the applicability of the DBRF-MEGN method to the large-scale expression profiles and the sensitivity of the DBRF-MEGN method to the noise of the expression profiles**. Click here for file

Additional file 3 **The MEGNs deduced from large-scale gene expression profiles**. Click here for file
