# Supplementary material for: A proof of the DBRF-MEGN method, an algorithm for deducing minimum equivalent gene networks
Source: Source Code Biol Med. 2011 Jun 24;6:12. doi: 10.1186/1751-0473-6-12 (PMC3152880; doi:10.1186/1751-0473-6-12)
Supplement: Additional file 1 — The complete source code files, a binary Linux executable file, and the software manual. [file 1751-0473-6-12-S1.ZIP › dbrf_megn/manual/manual_dbrf_megn.pdf]

DBRF-MEGN Method Manual

INTRODUCTION

The DBRF-MEGN (difference-based regulation finding - minimum equivalent gene network) method deduces gene regulatory networks from large-scale gene expression profiles of single deletion mutants. Each deduced gene regulation is represented as a signed directed edge whose sign – positive or negative – represents whether the effect of the regulation is activation or inhibition and whose direction represents which gene regulates which other gene. The method exactly deduces the most parsimonious signed directed graphs consistent with expression profiles of gene deletion mutants.

HARDWARE AND OS REQUIREMENT

The code of the DBRF-MEGN method was written by C++. Currently, the program of the DBRF-MEGN method runs under Linux platform on a typical IBM compatible personal computer. Although we do not confirm whether the program runs under the other platforms, it will run under the platforms that have standard C++ compiler (e.g., Windows, Solaris, Mac OS X).

INPUT FILE FORMAT

The program requires only one input file. Using the following sample, we show the format of the file. The first row represents a list of genes that are deleted. The first column represents a list of genes of which the expression levels are measured. The number and order of genes in the two lists must be the same. Each element of the remaining rows or columns represents gene expression ratio of deletant to wild-type. When there is not significant alteration of gene expression level between wild-type and deletant, the corresponding element must be set to be zero. Currently, the program only reads tab-delimited text files.

	gene_a	gene_b	gene_c	gene_d	gene_e	gene_f	gene_g
GENE_A	0	-1.1	-1.2	0	0	0	0
GENE_B	-1.1	0	-1.4	0	1.5	0	0
GENE_C	-1.5	-1.9	0	0	0	0	1.7
GENE_D	0	0	0	0	-1.2	-1.3	0
GENE_E	0	0	0	-1.1	0	-1.7	0
GENE_F	0	0	0	-1.2	-1.2	0	0
GENE_G	0	0	0	0	0	0	0

QUICK START

The program runs by using command "**dbrf_megn <data_file>**" from the command line. The *<data_file>* is the input file whose format is described above. When the MEGN consists only of the essential edges, the program generates only one file named by *essential_edges-<data_file>* that includes a list of the essential edges. When the MEGN consists of the essential edges and the restored edges, the program generates two files, one is named by *essential_edges-<data_file>*, which includes a list of the essential edges and another is named by *restored_edges-<data_file>*, which includes sets of the restored edges in independent groups.

The sample file *data_sample.txt* is included in the same directory or folder that includes the binary program of the DBRF-MEGN method. The program runs by the following command.

./dbrf_megn data_sample.txt

Then the program will generate two files, because the MEGN consists of the essential edges and the restored edges. One is *essential_edges-data_sample.txt* that includes a list of the essential edges and another is *restored_edges-data_sample.txt*, which includes sets of the restored edges in independent groups. The format of each of those files is described in the next section.

OUTPUT FILE FORMAT

The following sample is an example of the output file for the essential edges. Each row of the output file for the essential edges represents a deduced edge, which directs from the gene in the first column to that in the second column. The third column represents the sign of the edge (P: positive, N: negative).

from	to	effect
GENE_E	GENE_B	N
GENE_G	GENE_C	N

The following sample is an example of the output file for the restored edges. In the output file for the restored edges, there is more than one independent group of unexplained edges. The first row of the file indicates the number of independent groups. Combination of one set of those independent groups produce all MEGNs. In the following sample, 4 MEGNs are deduced.

num. of independent groups: 2

group 1(1 set out of 2 sets)

set	from	to	effect	from	To	effect	from	to	effect
1	GENE_A	GENE_B	P	GENE_B	GENE_C	P	GENE_C	GENE_A	P
2	GENE_A	GENE_C	P	GENE_B	GENE_A	P	GENE_C	GENE_B	P

group 2(1 set out of 2 sets)

set	from	to	effect	from	To	effect	from	to	effect
1	GENE_D	GENE_E	P	GENE_E	GENE_F	P	GENE_F	GENE_D	P
2	GENE_D	GENE_F	P	GENE_E	GENE_D	P	GENE_F	GENE_E	P
