# Supplementary material for: A proof of the DBRF-MEGN method, an algorithm for deducing minimum equivalent gene networks
Source: Source Code Biol Med. 2011 Jun 24;6:12. doi: 10.1186/1751-0473-6-12 (PMC3152880; doi:10.1186/1751-0473-6-12)
Supplement: Additional file 2 — Supporting text for the applicability of the DBRF-MEGN method to the large-scale expression profiles and the sensitivity of the DBRF-MEGN method to the noise of the expression profiles. [file 1751-0473-6-12-S2.DOC]

**Additional file 2:**

**Applicability of the DBRF-MEGN method to large-scale expression profiles**

In our previous study [14], we showed the applicability of the DBRF-MEGN method to actual large-scale expression profiles by applying it to a set of large-scale gene expression profiles for *Saccharomyces cerevisae* [7]. To strengthen the evidence of its applicability to large-scale expression profiles, we applied the method to another set of large-scale gene expression profiles obtained for *S. cerevisiae* [8]. The set of profiles comprises expression levels of 269 transcriptional factors measured in 269 gene deletants corresponding to those genes. Each expression level accompanies a P-value, which corresponds to the significance of the difference from the expression level in the wild-type [8]. Initially deduced edges were obtained at a wide range of the P-value thresholds of 0.01 to 0.05 (Additional file 2, Table S1; Additional file 3). With the P-value threshold of 0.01, the essential edges deductively explained the initially deduced edges. Essential edges failed to explain initially deduced edges when the threshold was 0.02, 0.03, 0.04, and 0.05. In these cases, the DBRF-MEGN method successfully deduced 3, 2, 32, and 32 MEGNs, respectively. In each of all cases, the computation took ~0.04 seconds. These results strengthen our previous conclusion that the method would most probably deduce MEGNs from most sets of expression profiles in an acceptable time [14].

**Sensitivity of the DBRF-MEGN method to the noise of the expression profiles**

To investigate the sensitivity of the DBRF-MEGN method to the noise of the expression profiles, we deduced MEGNs from a subset of large-scale gene expression profiles for *S. cerevisiae* [14,7] using various P-value thresholds [14]. The set of profiles comprises expression levels of 265 genes measured in 265 gene deletion mutants corresponding to those genes. Each expression level accompanies a P-value, which corresponds to the significance of the difference from the expression level in the wild-type. We considered the expression level in the deletion mutants to be increased (decreased) when the level significantly differed from that in the wild-type at a P-value less than a predefined threshold. The number of initially deduced edges increased as the threshold increased. Because the empirically determined optimal threshold is 0.01 [14], we assumed that the initially deduced edges were deduced without the influence of the noise of the profiles when threshold = 0.01 and that the noise increased/decreased the number of initially deduced edges when threshold $\neq$ 0.01. The MEGN deduced using threshold = 0.01 was used as the gold standard.

First, we decreased the threshold from 0.01 to 0.00166 to decrease the number of initially deduced edges. We found that the number of false-negative edges increased much faster than that of false-positive edges as the number of initially deduced edges decreased (Additional file 2, Table S2; Additional file 2, Figure S1a). The number of false-negative edges increased at a rate similar to the decreasing rate of the number of initially deduced edges, whereas the number of false-positive edges increased at a rate less than 30% of the decreasing rate of the number of initially deduced edges. The result indicates that the number of false-positive edges is less sensitive to the noise than that of false-negative edges when the noise decreases the number of initially deduced edges.

Second, we increased the threshold from 0.01 to 0.0252 to increase the number of initially deduced edges. In contrast to the result when decreasing the number of initially deduced edges, we found that the number of false-positive edges increased much faster than that of false-negative edges as the number of initially deduced edges increased (Additional file 2, Table S2; Additional file 2, Figure S1b). The number of false-positive edges increased at a rate similar to the increasing rate of the number of initially deduced edges, whereas the number of false-negative edges increased at a rate less than 30% of the increasing rate of the number of initially deduced edges. The result indicates that the number of false-negative edges is less sensitive to the noise than that of false-positive edges when the noise increases the number of initially deduced edges.

These results suggest the following guideline for the threshold: when the number of false-positive edges is more important than that of false-negative edges, the threshold should be a little lower than the optimal one; In contrast, when the number of false-negative edges is more important than that of false-positive edges, the threshold should be a little higher than the optimal one.

**Table S1 – Application to large-scale expression profiles of *S. cerevisiae*.**

The DBRF-MENG method was applied to a subset of large-scale gene expression profiles for *S. cerevisiae* [8] using various P-value thresholds (0.01~0.05). The threshold was increased and decreased from 0.01. IDE, ES, MEGN, #IG and #MEGN represent the numbers of initially deduced edges, essential edges in the MEGN, edges in the MEGN, independent groups, and MEGNs, respectively. TIME represents computation time (seconds).

| P-value | IDE | ES | MEGN | #IG | #MEGN | TIME (s) |
| --- | --- | --- | --- | --- | --- | --- |
| 0.01 | 611 | 525 | 525 | 0 | 1 | 0.020 |
| 0.02 | 925 | 740 | 741 | 1 | 3 | 0.025 |
| 0.03 | 1237 | 928 | 929 | 1 | 2 | 0.022 |
| 0.04 | 1524 | 1061 | 1067 | 2 | 32 | 0.037 |
| 0.05 | 1853 | 1205 | 1210 | 5 | 32 | 0.031 |

**Table S2 - Effect of the noise of expression profiles for the DBRF-MEGN method.**

The DBRF-MENG method was applied to a subset of large-scale gene expression profiles for *S. cerevisiae* [14,7] using various P-value thresholds [7]. The threshold was increased and decreased from 0.01. The MEGN at threshold = 0.01 was used as the gold standard. Dec. rate and Inc. rate reprepent decreasing and increasing rates of the initially deduced edges, respectively. IDE, MEGN, TP, FN, and FP represent the numbers of initially deduced edges, edges in the MEGN, true-positive, false-negative, and false-positive edges, respectively.

| P-value | Dec. rate | IDE | MEGN | TP | FN | FP |
| --- | --- | --- | --- | --- | --- | --- |
| 0.01 | 0 | 829 | 675 | 675 | 0 | 0 |
| 0.008 | 0.1 | 747 | 618 | 603 | 72 | 15 |
| 0.00577 | 0.2 | 664 | 568 | 529 | 146 | 39 |
| 0.00403 | 0.3 | 581 | 508 | 453 | 222 | 55 |
| 0.00285 | 0.4 | 498 | 441 | 382 | 293 | 59 |
| 0.00166 | 0.5 | 415 | 377 | 309 | 366 | 68 |
|  |  |  |  |  |  |  |
| P-value | Inc. rate | IDE | MEGN | TP | FN | FP |
| 0.01 | 0 | 829 | 675 | 675 | 0 | 0 |
| 0.0124 | 0.1 | 911 | 737 | 661 | 14 | 76 |
| 0.0154 | 0.2 | 994 | 794 | 646 | 29 | 148 |
| 0.0183 | 0.3 | 1077 | 846 | 625 | 50 | 221 |
| 0.0219 | 0.4 | 1160 | 894 | 600 | 75 | 294 |
| 0.0252 | 0.5 | 1243 | 932 | 577 | 98 | 355 |

**Figure S1 - Sensitivity of the DBRF-MEGN method to noise of the expression profiles**

(a) Ratios of the number of false-positive edges and those of the number of false-negative edges to the number of gold standard positive edges were plotted as a function of the decreasing rate of the number of initially deduced edges. (b) Ratios of the number of false-positive edges and those of the number of false-negative edges to the number of gold standard positive edges were plotted as a function of the increasing rate of the number of initially deduced edges. The number of initially deduced edges was increased or decreased from that at threshold = 0.01. The MEGN at threshold = 0.01 was used as the gold standard. FN, FP, and GSP represent the number of false-negative, false-positive, and gold standard positive edges, respectively.
